# Human Immunodeficiency Virus and Leprosy Coinfection: Challenges in Resource-Limited Setups

**DOI:** 10.1155/2012/698513

**Published:** 2012-05-09

**Authors:** Charles M. Kwobah, Kara K. Wools-Kaloustian, Jane N. Gitau, Abraham M. Siika

**Affiliations:** ^1^USAID-Academic Model Providing Access to Healthcare (AMPATH) partnership, P.O. Box 4606-30100, Eldoret, Kenya; ^2^Division of Infectious Diseases, Department of Medicine, Indiana University School of Medicine, Indianapolis, IN 46202-5124, USA; ^3^Department of Medicine, Moi University School of Medicine, P.O. Box 4606-30100, Eldoret, Kenya

## Abstract

*Mycobacteria leprae(leprosy)* and HIV coinfection are rare in Kenya. This is likely related to the low prevalence (1 per 10,000 of population) of leprosy. Because leprosy is no longer a public health challenge there is generally a low index of suspicion amongst clinicians for its diagnosis. Management of a HIV-1-leprosy-coinfected individual in a resource-constrained setting is challenging. Some of these challenges include difficulties in establishing a diagnosis of leprosy; the high pill burden of cotreatment with both antileprosy and antiretroviral drugs (ARVs); medications' side effects; drug interactions; scarcity of drug choices for both diseases. This challenge is more profound when managing a patient who requires second-line antiretroviral therapy (ART). We present an adult male patient coinfected with HIV and leprosy, who failed first-line antiretroviral therapy (ART) and required second-line treatment. Due to limited choices in antileprosy drugs available, the patient received monthly rifampicin and daily lopinavir-/ritonavir-based antileprosy and ART regimens, respectively. Six months into his cotreatment, he seemed to have adequate virological control. This case report highlights the challenges of managing such a patient.

## 1. Introduction

It is a well-established fact that in TB/HIV coinfected patients, each disease contributes to the progression of the other. Active TB in HIV-1-infected patients is associated with increased immunodeficiency and mortality [1–4]. It has been speculated that, as with TB, HIV infection may exacerbate leprosy lesions and/or lead to increased susceptibility to leprosy. However, there is no good evidence to support this speculation. Indeed, many studies have found that in coinfected patients, each disease progresses independently [5–8].

Treatment of an HIV-1-leprosy coinfected patient requires a potent combination of ARVs as well as antileprosy agents. The ART must be taken with strict adherence to achieve maximal suppression of viral replication. This in turn limits the risk of developing drug resistance [9, 10]. Achieving high adherence levels in coinfected patients may be challenging due to the high pill burden.

We report the case of a patient coinfected with HIV-1 and leprosy who developed virological failure to first-line ART. He was subsequently switched to second-line ART. Six months later, antileprosy chemotherapy was initiated.

## 2. Case Report

A 66-year-old male was seen in a USAID, Academic Model Providing Access to Healthcare (AMPATH) Partnership clinic in western Kenya, complaining of general body itchiness and skin patches on the chest for 3 months prior to contact. He had tested HIV positive three months prior to enrollment at the clinic. He complained of poor appetite and had lost 3 kg. He did not report any allergies to food or drugs, did not smoke, but reported that he takes alcohol (local brew) 2-3 times/week.

On physical examination, he was in fair general condition with a BP of 100/60 mmHg, pulse rate of 92 beats per minute, temperature 36.1°C, and arterial oxygen saturation of 93% while breathing ambient air. He weighed 65 kgs. His skin had 3 hypopigmented and hypoaesthetic patches on the right chest wall and right upper arm. The largest lesion measured 5 by 6 cm and the smallest 2 by 3 cm. Other systems were essentially normal. Baseline investigations revealed a CD4 cell count of 145/mL, CD4 percentage 10%, white blood cell count of 2800/uL, Hemoglobin 12.3 g/dL, and platelets count of 178,000/uL. SGPT and creatinine were within normal ranges. His chest X-ray was without evidence of pathology.

An impression of *Tinea corporis* was made and the patient assigned WHO clinical stage 2 due to weight loss of <10%. He was given cotrimoxazole prophylactic therapy and clotrimazole cream. One month later, the patient was started on an ART regimen composed of stavudine, lamivudine, and nevirapine after adherence counseling. The patient's progress (CD4 counts, viral load and weight) is summarized in Table 1.

The patient had poor adherence based upon missed clinic appointments and self report. There is documented history that the patient did not take his medications for a period of one month during the 3rd year of ART. He attributed this to social stressors. An outreach worker had to visit his home to remind him of clinic visits. After several adherence counseling sessions, his plasma viral load dropped from 1,084,798/mL at 29 months of treatment to 433,376/mL six months later. At this point, virological treatment failure was confirmed based on a plasma viral load of >5000 copies/mL. A decision was made to continue with first-line ART until perfect adherence is maintained for at least six months before initiating second-line ART. All this time the skin lesions persisted despite regular use of clotrimazole cream.

After demonstrating good adherence, the patient was initiated on second-line ART regimen including abacavir, didanosine, and aluvia (Lopinavir/Ritonavir) 47 months after initiating first-line ART. The skin lesions persisted but did not increase in number or size. A decision to refer him to the regional center for leprosy was made where a skin smear was done and was positive for acid fast bacilli. According to Ridley-Jopling system, he was classified as having borderline tuberculoid leprosy based on having three lesions only that were well demarcated, asymmetrical, and unilateral. However, there was no enlargement of the nerve trunks and no neurological deficits other than the hypoaesthesia. According to the WHO classification, he was classified as having multibacillary leprosy based on having a positive skin smear on at least one site. Seven months after initiating second-line ART, he was put on rifampicin 600 mg monthly, clofazimine 300 mg once a month and 50 mg daily and dapsone 100 mg daily for 12 months which is the WHO recommended multidrug treatment (MDT) regimen for multibacillary leprosy. Six months later, the skin lesions had resolved, there was no neurological sequelea, and a check viral load was 188 copies/mL.

Eighteen months since initiating MDT for leprosy, the patient remained stable without new lesions, nor neurological deficits. A follow-up skin biopsy showed mild chronic inflammation with noncaseating granulomas. Fite's Acid fast stain for leprosy was negative. Genotypic viral resistance testing performed revealed resistance mutations to nonnucleotide reverse transcriptase inhibitors (NNRTI's) and nucleoside reverse transcriptase inhibitors (NRTI's) but none to protease inhibitors (PI's).

## 3. Discussion: Leprosy in HIV-1 Disease

Globally, the new case detection rate of leprosy is generally declining. The registered prevalence rate as of 2011 was highest in the South East Asia (0.64/10,000 of the population) followed by the African region (0.38/10,000 of the population). In Africa, the highest number of new infections in 2010 was reported in the Democratic Republic of Congo followed by Ethiopia (5,049 and 4,430, resp.) [11]. In Kenya, the number of new leprosy cases detected decreased from 630 in 1986 to 157 in 2009. Kenya is in the postelimination phase of leprosy and thus HIV-1-leprosy coinfection is rare [12].

This is a case of *M.leprae*-HIV true coinfection according to Talhari et al. classification [13]. This classification recognizes true leprosy-HIV-coinfection, opportunistic leprosy disease, and leprosy related to ART. To the best of our knowledge, this is the first case of second-line ART in a patient on treatment for leprosy reported in Kenya. This case illustrates the challenges that face clinicians in the diagnosis of leprosy in HIV-1-infected patients in low-resource setups. In illustration, the diagnosis of leprosy was not thought of for several years since his first presentation to clinic. This led to the patient being treated for a presumed fungal skin infection for a long time before the definitive diagnosis was made. We postulate that the rarity of leprosy in Kenya has led to a low index of suspicion for the disease amongst clinicians. Also, very few clinicians in Kenya have had clinical exposure to leprosy. This means that few have the clinical experience to recognize the lesions. In addition, a definitive diagnosis of leprosy requires microscopic examination of skin specimens, a tool that is not available in majority of facilities in Kenya.

The skin lesions of leprosy are classified morphologically according to the Ridley-Jopling classification system into five groups based on the immunity [14]. Patients with a strong immune system have tuberculoid leprosy in which the skin lesions are usually hypopigmented, hypoaesthetic, welldefined with clear margins. They are usually asymmetric and less than five in number. The nerve trunks tend to enlarge and become palpable and may lead to significant neurological deformities. Patients with a poor immune response develop lepromatous leprosy which is characterized by numerous papules and nodules which are distributed symmetrically. In between these poles is a spectrum that includes borderline tuberculoid, borderline, and borderline lepromatous in ascending order of severity. Presence of hypopigmented hypoaesthetic skin lesions should prompt the physician to consider a diagnosis of leprosy in a patient.

As noted earlier, HIV-1 does not seem to affect the clinical classification and progression of leprosy. In a study by AS Pereira et al. [15] the clinical, immunologic, histopathologic, and virologic features among 22 HIV-1-leprosy-coinfected Brazilian patients indicate that each disease progressed as in single infection [15]. Despite overall HIV-associated immunosuppression, cell-mediated immune responses to *M. leprae* are well preserved at the site of the disease [16]. In our patient, the disease progressed slowly, and the lesions did not alter morphologically over a period of 4 years of followup. This suggests that the pathogenesis of leprosy in this patient was unaffected by his HIV-1-related immunodeficiency.

The use of rifampicin in a patient on protease inhibitor-based ART is problematic because of the potential for drug interactions. Rifampicin is a potent inducer of the cytochrome P450-3A4 subenzyme, which is responsible for the metabolism of protease inhibitors (PI), amongst other drugs [17, 18]. This may result in subtherapeutic concentrations of the PI thus increasing the risk of treatment failure and virological resistance. Studies have shown that boosting a PI like saquinavir with ritonavir, a strong inhibitor of CYP3A4, may allow the coadministration of rifampicin. However, the use of this regimen is limited by adverse events due to higher doses of ritonavir (400 mg) used for boosting. This has been associated with increased hepatotoxicity, nausea, and vomiting [19–22]. The most widely available PI in Kenya and in most African countries is ritonavir-boosted lopinavir. Coadministration of rifampicin with standard dose lopinavir/ritonavir (400 mg/100 mg) leads to a subtherapeutic concentration of the latter. However, an adjusted dose of lopinavir/ritonavir (800 mg/200 mg or 400 mg/400 mg) in combination with therapeutic drug monitoring and monitoring of liver function may allow concomitant use of rifampicin in healthy volunteers. However, this regimen is not well tolerated because of increased incidence of nausea and vomiting [23].

In our patient, the treatment of leprosy required administration of rifampicin 600 mg once a month. We presumed that monthly rifampicin would have minimal interactions with the lopinavir-/ritonavir-based ART. As such, we did not adjust the dose of lopinavir/ritonavir in the realization that such an adjustment would only serve to increase toxicity. Thus the patient received standard dose lopinavir/ritonavir together with monthly rifampicin. This combination was well tolerated and resulted in good outcome for both diseases. The skin lesions resolved within six months of treatment and the viral load revealed good virological control.

 It is recommended that rifabutin should substitute rifampicin in the treatment of TB in TB/HIV-1-coinfected patients on PI-based ART [24, 25]. However, for the treatment of leprosy, there is limited data to support the use of rifabutin in HIV-1-leprosy-coinfected patients. Earlier preclinical studies in mice and armadillos suggested that rifabutin can be a substitute for rifampicin in the multidrug treatment of leprosy [26, 27]. In addition, a number of in-vitro studies have demonstrated the potency of rifabutin against *M.leprae* [28–30]. However, there is a paucity of data from clinical trials that evaluate the efficacy of rifabutin in the multidrug treatment of leprosy in HIV-1-coinfected patients. Similarly, the substitution of rifampicin with a floroquinolone in the multi-drug therapy for leprosy has not been well tested in this population, if at all.

Our management of this patient was informed by the fact that there is no clinically validated substitute for rifampicin in the treatment of leprosy in HIV-1-coinfected patients. In addition to that, we did not have an alternative to lopinavir/ritonavir for the treatment of HIV. Though we were unable to monitor the drug concentrations in his blood due to resource limitation, surrogate markers such as the clinical, immunological, and virological parameters suggested a good response.

 Initiation of ART has been associated with Immune Reconstitution and Inflammatory Syndrome (IRIS) in various situations. IRIS in leprosy may trigger potential adverse effects, such as leprosy acute inflammatory episodes [31–34]. This usually leads to a worsening of the initial lesion characterized by erythema and tenderness in the setting of rising CD4 count and falling viral load. These reactions are more common in patients with low CD4 counts especially during the initial 3 months of initiation of ART. Typically, as the immune system further recovers, the lesions become tuberculoid/paucibacillary as opposed to lepromatous. In our patient, there was no change in the appearance of the skin lesions after starting both first-line and second-line ART, despite evident virological suppression and immune reconstitution with the latter. More so, there were no neurological deficits noted even after initiating potent second-line ART.

The follow-up skin biopsy revealed mild chronic dermatitis, but Fite's Acid fast stain was negative, suggesting that the patient is cured of leprosy. The absence of PI resistance mutations suggests that the dosing schedule of rifampicin adopted in this patient did not adversely affect the efficacy of the PI-based regimen. This, however, must be interpreted with caution since it is known that PI's have a high genetic barrier to resistance.

## 4. Conclusion

Standard dose lopinavir-/ritonavir-based ART coadministered with monthly rifampicin may still achieve virological suppression in HIV-1-leprosy coinfected patients.

## Figures and Tables

**Table 1:** Clinical, immunological, and virological parameters of an HIV-1-leprosy coinfected patient in western Kenya.

EVENT	Weight (kg)	CD4 count (Cells/mL)	CD4%	Plasma viral load (copies/mL)
Enrolment	65	145	10	
ART initiated 1 month later				
5 months*	66	272	16	
12 months*	64	154	13	
15 months*	65	152	13	
23 months*	62	192	11	
27 months*	58	110	8	
29 months*				1,084,798
32 months*	62	169	11	
35 months *				433,376
40 months*	60	155	13	
45 months*	55	195	7	
47 months: Switch to second-line ART				
6 months**	60	320	11	
7 months**	Started antileprosy chemotherapy			
13 months**				188

*After ART initiation.

**After initiation of second-line ART.

## References

[1] Toossi Z, Mayanja-Kizza H, Hirsch CS (2001). Impact of tuberculosis (TB) on HIV-1 activity in dually infected patients. *Clinical and Experimental Immunology*.

[2] Falvo JV, Ranjbar S, Jasenosky LD, Goldfeld AE (2011). Arc of a vicious circle: pathways activated by Mycobacterium tuberculosis that target the HIV-1 long terminal repeat. *American Journal of Respiratory Cell and Molecular Biology*.

[3] CDC (2010). HIV testing and treatment among tuberculosis patients—Kenya 2006–2009. *Morbidity and Mortality Weekly Report (MMWR)*.

[4] Whalen C, Horsburgh CR, Hom D, Lahart C, Simberkoff M, Ellner J (1995). Accelerated course of human immunodeficiency virus infection after tuberculosis. *American Journal of Respiratory and Critical Care Medicine*.

[5] Ustianowski AP, Lawn SD, Lockwood DN (2006). Interactions between HIV infection and leprosy: a paradox. *The Lancet Infectious Diseases*.

[6] Munyao TM, Bwayo JJ, Owili DM, Ndinya-Achola JO, Kwasa TO, Kreiss JK (1994). Human immunodeficiency virus- 1 in leprosy patients attending Kenyatta National Hospital, Nairobi.. *East African Medical Journal*.

[7] Sarno EN, Illarramendi X, Costa Nery JA (2008). HIV-M. leprae interaction: can HAART modify the course of leprosy?. *Public Health Reports*.

[8] Lockwood DNJ, Lambert SM (2011). Human immunodeficiency virus and leprosy: an update. *Dermatologic Clinics*.

[9] Harrigan PR, Hogg RS, Dong WWY (2005). Predictors of HIV drug-resistance mutations in a large antiretroviral-naive cohort initiating triple antiretroviral therapy. *Journal of Infectious Diseases*.

[10] Tam LWY, Chui CKS, Brumme CJ (2008). The relationship between resistance and adherence in drug-naive individuals initiating HAART is specific to individual drug classes. *Journal of Acquired Immune Deficiency Syndromes*.

[11] wer8636.pdf http://www.who.int/wer/2011/wer8636.pdf.

[12] Ministry of Health http://www.nltp.co.ke/tbleprosy.html.

[13] Talhari C, Mira MT, Massone C (2010). Leprosy and HIV coinfection: a clinical, pathological, immunological, and therapeutic study of a cohort from a brazilian referral center for infectious diseases. *Journal of Infectious Diseases*.

[14] Ridley DS, Jopling WH (1966). Classification of leprosy according to immunity. A five-group system.. *International Journal of Leprosy and Other Mycobacterial Diseases*.

[15] Pereira GAS, Stefani MMA, Araújo Filho JA, Souza LCS, Stefani GP, Martelli CMT (2004). Human immunodeficiency virus type 1 (HIV-1) and Mycobacterium leprae co-infection: HIV-1 subtypes and clinical, immunologic, and histopathologic profiles in a Brazilian cohort. *American Journal of Tropical Medicine and Hygiene*.

[16] Massone C, Talhari C, Talhari S (2011). Immunophenotype of skin lymphocytic infiltrate in patients co-infected with Mycobacterium leprae and human immunodeficiency virus: a scenario dependent on CD8+ and/or CD20+ cells. *British Journal of Dermatology*.

[17] Kolars JC, Schmiedlin-Ren P, Schuetz JD, Fang C, Watkins PB (1992). Identification of rifampin-inducible P450IIIA4 (CYP3A4) in human small bowel enterocytes. *Journal of Clinical Investigation*.

[18] Finch CK, Chrisman CR, Baciewicz AM, Self TH (2002). Rifampin and rifabutin drug interactions: an update. *Archives of Internal Medicine*.

[19] Veldkamp AI, Hoetelmans RMW, Beijnen JH, Mulder JW, Meenhorst PL (1999). Ritonavir enables combined therapy with rifampin and saquinavir. *Clinical Infectious Diseases*.

[20] CDC (2000). Updated guidelines for the use of rifabutin or rifampin for the treatment and prevention of tuberculosis among HIV-infected patients taking protease inhibitors or nonnucleoside reverse transcriptase inhibitors. *Morbidity and Mortality Weekly Report (MMWR)*.

[21] Rolla VC, da Silva Vieira MA, Pinto DP (2006). Safety, efficacy and pharmacokinetics of ritonavir 400mg/saquinavir 400mg twice daily plus rifampicin combined therapy in HIV patients with tuberculosis. *Clinical Drug Investigation*.

[22] HIV and AIDS Activities Drug Interaction Warning: saquinavir/ritonavir and Rifampin. http://www.fda.gov/ForConsumers/ByAudience/ForPatientAdvocates/HIVandAIDSActivities/ucm124918.htm.

[23] La Porte CJL, Colbers EPH, Bertz R (2004). Pharmacokinetics of adjusted-dose lopinavir-ritonavir combined with rifampin in healthy volunteers. *Antimicrobial Agents and Chemotherapy*.

[24]  Loeliger A, Suthar AB, Ripin D (2012). Protease inhibitor-containing antiretroviral treatment and tuberculosis: can rifabutin fill the breach?. *The International Journal of Tuberculosis and Lung Disease*.

[25] Breen RAM, Swaden L, Ballinger J, Lipman MCI (2006). Tuberculosis and HIV co-infection: a practical therapeutic approach. *Drugs*.

[26] Dhople AM, Ibanez MA (1994). In vivo susceptibility of Mycobacterium leprae to ofloxacin either singly or in combination with rifampicin and rifabutin. Anti-leprosy activity of ofloxacin and ansamycins in mice. *Arzneimittel-Forschung*.

[27] Dhople AM, Williams SL (1998). The activity of rifabutin against Mycobacterium leprae in armadillos. *International Journal of Antimicrobial Agents*.

[28] Yoder LJ, Jacobson RR, Hastings RC (1991). The activity of rifabutin against Mycobacterium leprae. *Leprosy Review*.

[29] Dhople AM, Ibanez MA, Gardner GD (1993). In vitro synergistic activity between ofloxacin and ansamycins against Mycobacterium leprae. *Arzneimittel-Forschung*.

[30] Ramasesh N, Krahenbuhl JL, Hastings RC (1989). In vitro effects of antimicrobial agents on Mycobacterium leprae in mouse peritoneal macrophages. *Antimicrobial Agents and Chemotherapy*.

[31] Massone C, Talhari C, Ribeiro-Rodrigues R (2011). Leprosy and HIV coinfection: a critical approach. *Expert Review of Anti-Infective Therapy*.

[32] Kharkar V, Bhor UH, Mahajan S, Khopkar U (2007). Type I lepra reaction presenting as immune reconstitution inflammatory syndrome. *Indian Journal of Dermatology, Venereology and Leprology*.

[33] Chow D, Okinaka L, Souza S, Shikuma C, Tice A (2009). Hansen’s disease with HIV: a case of immune reconstitution disease.. *Hawaii Medical Journal*.

[34] Kar HK, Sharma P (2007). Dose concomitant HIV infection has any epidemiological, clinical, immunopathological and therapeutic relevance in leprosy?. *Indian Journal of Leprosy*.

